# Association between Galectin-3 levels within central and peripheral venous blood, and adverse left ventricular remodelling after first acute myocardial infarction

**DOI:** 10.1038/s41598-019-49511-4

**Published:** 2019-09-11

**Authors:** Olivera M. Andrejic, Rada M. Vucic, Milan Pavlovic, Lana McClements, Dragana Stokanovic, Tatjana Jevtovic–Stoimenov, Valentina N. Nikolic

**Affiliations:** 1 Clinic for Pulmonary Diseases, Clinical Centre Kragujevac, Zmaj Jovina Street 30, 34000 Kragujevac, Serbia; 2 Department of Internal medicine, Faculty of Medical Sciences, University of Kragujevac, Serbia, Svetozara Markovica Street 69, 34000 Kragujevac, Serbia; 3 Clinic for Cardiovascular Diseases, Clinical Centre Kragujevac, Zmaj Jovina Street 30, 34000 Kragujevac, Serbia; 4 Department of Internal Medicine - Cardiology, Medical Faculty, University of Nis, Bulevar dr Zorana Djindjica 81, Nis, Serbia; 5 Clinic for Cardiovascular Diseases, Clinical Centre Nis, Bulevar dr Zorana Djindjica 48, Nis, Serbia; 6 School of Life Sciences, Faculty of Science, University of Technology Sydney, Sydney, PO Box 123, Broadway, NSW 2007 Australia; 7 Department of Pharmacology and Toxicology, Medical Faculty, University of Nis, Bulevar dr Zorana Djindjica 81, Nis, Serbia; 8 Institute of Biochemistry, Medical Faculty, University of Nis, Bulevar dr Zorana Djindjica 81, Nis, Serbia

**Keywords:** Cardiology, Physiology

## Abstract

Our study investigates association between Galectin-3 levels and adverse left ventricular remodelling (LVR) at six months. Fifty-seven patients following first acute myocardial infarction (AMI) were enrolled in this study and blood samples collected on day 1 from the femoral vein and artery, the right atrium near the coronary sinus and the aortic root, and on day 30, from the cubital vein. Patients with LVESV ≥20% at six months, were included in the LVR group. On day 1, Galectin-3 plasma levels in the femoral vein (10.34 ng/ml ± 3.81 vs 8.22 ng/ml ± 2.34, p = 0.01), and near coronary sinus (10.7 ng/ml ± 3.97 vs 8.41 ng/ml ± 2.56, p = 0.007) were higher in the LVR group. Positive correlations between Galectin-3 levels from aortic root and coronary sinus, aortic root and femoral vein, and coronary sinus and femoral vein, were observed in both groups. On day 30, Galectin-3 concentration in the cubital vein was an independent risk factor of LVR six months post-AMI, demonstrating 1.5-fold increased risk. Day-30 Galectin-3 also showed positive correlations with echocardiography parameters indicative of diastolic and systolic dysfunction. Determining Galectin-3 plasma concentration on day 30 following AMI could have beneficial prognostic value in predicting LVR.

## Introduction

Left ventricular remodelling (LVR) is a set of changes in the ventricular structure and function following acute myocardial infarction (AMI), associated with a progressive increase in left ventricular end-systolic volume (LVESV) and left ventricular end-diastolic volume (LVEDV). These changes can lead to deterioration of the left ventricular systolic function measured by left ventricle ejection fraction (LVEF) and further cardiovascular complications^1,2^. Structural changes of the left ventricle occur in the necrotic area of the infarcted myocardium, border zone and remote zone in the non-infarcted myocardium. These changes are characterized by an increase in the myocardial infarction area, collagen deposits, scar formation, and hypertrophy of the non-infarcted myocardium. Initial increase in collagen and formation of fibrotic tissue lead to a decrease in ventricular wall tension and aid preservation of the shape and contractile function of the left ventricle in the early period following AMI, but excessive and prolonged collagen production post-AMI is associated with adverse LVR^3^.

Following AMI, local and systemic pro-inflammatory factors have an important role in LVR^4^. Increased collagen production leading to fibrosis, is a result of cardiac fibroblasts activation in response to mediators such as transforming growth factor β (TGF-β) or activation of the renin-angiotensin-aldosterone system^4^. Galectin-3 is another mediator which has a role in the LVR process. High levels of Galectin-3 have been detected in activated macrophages following myocardial injury, and its role in stimulating cardiac fibroblasts to synthesize collagen type I is well-established^5^. High systemic and cardiac levels of Galectin-3 have been positively correlated with the number of infiltrating macrophages and deposition of extracellular matrix^5,6^. The pro-inflammatory and pro-fibrotic protective roles of Galectin-3, in the early phase of AMI, play an important role in myocardium tissue repair^7^. However, continuous and excessive activation of inflammation and fibrosis, along with high levels of Galectin-3, are associated with LVR and poor clinical outcomes^8–11^. Therefore, determining the localization and timing of the positive and negative prognostic value of Galectin-3 in terms of LVR post-AMI is key.

Previous studies have investigated the LVR prognostic value of circulating Galectin-3 levels following AMI using blood samples collected from a peripheral vein with conflicting results^12,13^. Therefore, the aim of our study was to investigate association between circulating Galectin-3 levels within central and peripheral arterial and venous blood on day 1 and peripheral venous blood on day 30, post-AMI, in short to medium-term LVR. We also present detailed analysis of correlations between Galectin-3 levels from each sampling locations as well as correlations between Galectin-3 levels and left ventricular parameters at day 1, 30 and six months after AMI.

## Results

### Clinical characteristics of patients with and without LVR

Six months after AMI, 22 patients with an average age of 62.55 ± 9.10 years experienced LVR, whereas 35 patients did not (age: 63.37 ± 10.03). There were no differences in demographic characteristics or the vast majority of the co-morbidities between the groups, except for the initial higher frequency of diabetes mellitus in the group of patients who developed LVR six months after AMI (7 vs 13 patients, p < 0.001; Table 1). Patients who experienced LVR had a higher leukocyte count (9.5 vs 9.0 (×10^12^/L), p = 0.02) and CRP (13.5 vs 3.4 (mmol/l), p = 0.03) at baseline. There were no significant differences in the clinical presentation of AMI and coronary artery infarction lesion localization, between the two groups during initial hospitalization (Table 1).

**Table 1:** Baseline characteristics of patients with and without adverse left ventricle remodeling.

Variables	No LVR (n = 35)	LVR (n = 22)	t* or Z** or χ^2^***	p value
Gender (male)	27 (79.40%)	16 (72.70%)	0.355***	0.563
Age (years)	63.37 ± 10.03	62.55 ± 9.10	0.313*	0.755
Time of pain onset (hours)	10.00 (3.00–20.00)	14.00 (7.00–20.5)	1.709**	0.089
Diabetes mellitus	7 (20%)	13 (59%)	**9**.063***	**0**.**000**
Atrial fibrillation	3 (8.6%)	2 (9.1%)	0.000***	1.000
Arterial hypertension	21 (60.0%)	15 (68.2%)	0.389***	0.553
Hyperlipoproteinemia	13 (37.1%)	6 (27.3%)	0.592***	0.442
Smoking habit	13 (62.80%)	8 (63.6%)	0.004***	0.953
BMI(kg/m^2^)	27.75 ± 3.7	28.57 ± 3.01	0.888*	0.378
Diastolic Blood Pressure (mmHg)	78.40 ± 14.60	72.95 ± 19.25	1.211*	0.231
Systolic Blood Pressure (mmHg)	133.28 ± 23.51	125.59 ± 33.54	1.018*	0.313
Heart Rate (bpm)	75.09 ± 13.81	74.23 ± 15.45	0.218*	0.828
STEMI	22 (62.9%)	13 (59.1%)	0.081***	0.780
AV block	1 (2.9%)	2 (9.1%)	0.174***	0.553
VF/VT	5 (14.3%)	4 (9.1%)	0.028***	0.695
Artery with culprit lesion	12 (37.1%)	10 (45.5%)	3.570***	0.312
LAD stenosis (%)	60.00 (50.00–100.00)	42.50 (0.00–99.25)	0.008**	0.993
LCx stenosis (%)	70.00 (50.00–99.00)	70.00 (0.00–75.00)	1.008**	0.313
RCA stenosis (%)	60.00 (40.00–80.00)	99.50 (75.00–100.00)	1.290**	0.197
One-vessel disease	6 (17.10%)	6 (27.30%)	0.336***	0.506
Two-vessel disease	16 (45.70%)	5 (22.70%)	3.086***	0.080
Multi-vessel disease	15 (42.90%)	11 (50.00%)	0.278***	0.598
Total Cholesterol (mmol/L)	5.76 ± 1.13	5.84 ± 1.03	0.268*	0.790
Triglycerides (mmol/L)	1.15 (0.91–1.71)	1.76 (1.37–2.04)	0.520**	0.603
LDL (mmol/L)	3.71 ± 1.05	3.73 ± 1.004	0.076*	0.940
HDL (mmol/L)	1.12 ± 0.24	1.06 ± 0.278	0.804*	0.425
Leukocyte count (x10^12^/L)	9.0 (8.30–11.70)	9.5 (9.17–12.60)	**2**.404**	**0**.**016**
Haemoglobin (g/L)	142.00 (132–142)	130.50 (112.50–154.50)	0.847**	0.397
hsCRP (mg/L)	3.4 (1.35–7.30)	13.50 (4.70–87.20)	**2**.112**	**0**.**035**
proBNP (pg/ml)	250.00 (179–2,071)	1,577 (309.50–4,926)	1.330**	0.182
Creatinine (μmol/L)	87.5 (78.0–99.8)	86.0 (77.5–109.5)	0.025*	0.980
Creatine clearance (ml/min)	82 ± 27.28	88 ± 38.20	0.586*	0.560
Glucose (mmol/l)	6.00 (5.10–8.10)	5.50 (4.63–9.60)	0.635**	0.525
TnT	1.2 (0.7–10.26)	0.51 (0.13–3.08)	0.825**	0.409
CKMB	27 (20–69.5)	20.5 (13–30)	1.109**	0.268
Nitrates	12 (34.3%)	9 (40.9%)	0.255***	0.614
Furosemide	11 (31.4%)	5 (22.7%)	0.507***	0.447
Spironolactone	7 (20.0%)	4 (18.2%)	0.000***	1.000
ACE inhibitors	26 (74.3%)	18 (81.8%)	0.435***	0.509
Beta blocker	26 (74.3%)	19 (86.4%)	0.570***	0.335
Calcium channel blockers	3 (8.6%)	4 (18.2%)	0.438***	0.411
Proton pump inhibitors	15 (42.9%)	13 (59.1%)	1.424***	0.233
H2 blockers	8 (22.9%)	2 (9.1%)	0.946***	0.278
Amiodarone	8 (22.9%)	5 (22.7%)	0.000***	0.991
Dual antiplatelet therapy	32 (91.4%)	21 (95.5)	0.002***	1.000
Ticagrelor	20 (57.1%)	13 (59.1%)	0.021***	0.885
Trimetazidine	12 (34.3%)	12 (54.5%)	2.270***	0.132
Statins	33 (94.3%)	21 (95.5%)	0.000***	1.000

All values are presented as mean ± SD or median with interquartile range (IQR) or numbers (%).

Two-tailed unpaired t-test (normalized distribution; t(p)) or Man-Whitney (non-normalized distribution; Z (p)), and χ^2^ (p).

LVR – left ventricular remodelling, STEMI - ST elevation myocardial infarction, AV - atrioventricular, VT/VF- ventricular tachycardia/ventricular fibrillation, BMI- body mass index, LMCA – left main coronary artery disease, LAD - left anterior descendent artery, LCx - left circumflex artery, RCA - right coronary artery, CRP - C reactive protein, proBNP - pro brain natriuretic peptide, TnT-Troponin T, CKMB-creatine kinase isoenzime MB, ACE inhibitor -angiotensine converting enzyme inhibitor.

### Association between Galectin-3 levels and left ventricular remodelling

Galectin-3 plasma levels were higher in patients with LVR in the femoral vein, right atrium near coronary sinus, on the first day of AMI (femoral vein: 10.34 ng/ml ± 3.81 vs 8.22 ng/ml ± 2.34, p = 0.01; coronary sinus: 10.71 ng/ml ± 3.97 vs 8.41 ng/ml ± 2.56, p = 0.007; Table 2), and in the cubital vein at day 30 (10.41 ng/ml ± 4.03 vs 7.28 ng/ml ± 2.85, p = 0.007; Table 2). No differences were observed in arterial Galectin-3 levels between patients with and without LVR.

**Table 2:** Galectin 3 plasma concentration (ng/ml) in patients with and without adverse left ventricular remodeling.

Variables	No LVR (n = 35)	LVR (n = 22)	t value	p-value
Aortic Root day 1	9.55 ± 5.65	10.22 ± 3.67	0.425	0.693
Femoral Artery day 1	9.29 ± 3.36	10.83 ± 4.29	1.32	0.194
Femoral Vein day 1	8.22 ± 2.34	10.34 ± 3.81	**2**.**589**	**0**.**012**
Right Atrium near the Coronary Sinus day 1	8.41 ± 2.56	10.71 ± 3.97	**2**.**803**	**0**.**007**
Cubital Vein day 30	7.28 ± 2.85	10.41 ± 4.03	**2**.**775**	**0**.**007**

All values are presented as mean ± SD.

Two-tailed unpaired t-test (normalized distribution; t(p)).

LVR – left ventricular remodeling.

**Figure 1:**
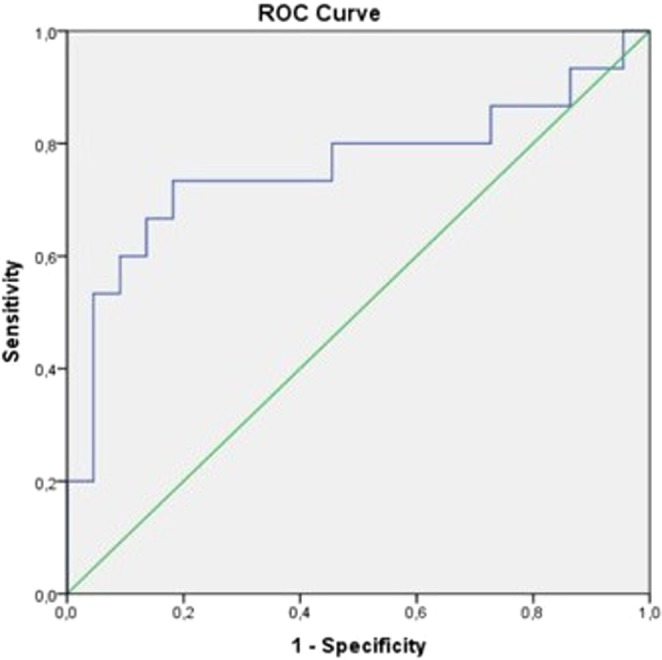
ROC curves for predicting the risk of developing adverse left ventricular remodelling based on Galectin-3 concentrations in the median cubital vein on day 30.

Using multivariate logistic regression modelling, we identified two variables as potential determinants of LVR six months after AMI. At day 30 after AMI, an increase in Galectin-3 plasma concentration in the median cubital vein of 1 unit, was independently associated with the 1.55-fold (p = 0.01) increased risk of LVR, six months after AMI (Table 3), adjusted for age, leukocyte count, CRP and diabetes. Diabetes was a very strong predictor of LVR in our study (OR = 68.2, p = 0.004; Table 3). Galectin-3 concentrations in the right atrium near the coronary sinus on day 1 and in the median cubital vein on day 30 showed the most promising sensitivity and specificity, based on the receiver operating characteristics (ROC) curves, for predicting the risk of developing LVR. Galectin-3 concentration in the right atrium near the coronary sinus on day 1, at the cut-off value of 9.42 ng/ml (AUC = 0.691, p = 0.02) showed sensitivity of 66.70% and specificity of 76.47% (Supplementary Fig. 1A) whereas the ROC curves in relation to other blood sampling locations on day 1 did not provide significance or satisfactory sensitivity or specificity (Supplementary Fig. 1B–D). More clinically relevant, Galectin-3 concentration in the median cubital vein on day 30, based on the cut-off value of 8.87 ng/ml, which was associated with sensitivity of 73.33% and specificity of 81.82% (AUC = 0.758, p = 0.006: Fig. 1) for predicting the risk of developing LVR.

**Table 3:** Multivariate logistic regression analysis of variable of adverse left ventricular remodelling six months after acute myocardial infarction (adjusted for age, leukocyte count and CRP).

Variable	Adjusted odds ratio (95% CI)	p-value
Diabetes mellitus	68.192 (3.872–1200.838)	0.004
Galectin-3 on day 30	1.554 (1.106–2.183)	0.011

### Correlations between Galectin-3 levels from different sampling locations

In the group of patients with adverse LVR, we observed positive correlation between Galectin-3 plasma concentration in the aortic root and femoral vein (r = 0.947, p < 0.001), aortic root and coronary sinus (r = 0.945, p < 0.001), and coronary sinus and femoral vein (r = 0.933, p < 0.001; Table 4) on day 1. Similar but weaker correlations were also found in patients without LVR, between Galectin-3 concentrations in aortic root and femoral vein (r = 0.436, p < 0.05), aortic root and coronary sinus (r = 0.465, p < 0.01), and coronary sinus and femoral vein (r = 0.532, p < 0.001, Table 5).

**Table 4:** Correlation between plasma Galectin-3 levels from different locations and time of sample tooking in patients with adverse LVR.

Variables		Coronary sinus Day 1	Femoral artery Day 1	Femoral vein Day 1	Median cubital vein Day 30
Aortic root Day 1	r	**0**.**945**	0.084	**0**.**947**	0.042
	p	**0**.**000**	0.757	**0**.**000**	0.882
Coronary sinus Day 1	r		0.084	**0**.**933**	0.139
	p		0.768	**0**.**000**	0.636
Femoral artery Day 1	r			0.272	−0.176
	p			0.308	0.585
Femoral vein Day 1	r				0.059
	p				0.835

**Table 5:** Correlation between plasma Galectin-3 levels from different locations and time of sample tooking in patients without LVR.

Variables		Coronary sinus Day 1	Femoral artery Day 1	Femoral vein Day 1	Median cubital vein Day 30
Aortic root Day 1	r	**0**.**465**	0.007	**0**.**436**	−0.035
	p	**0**.**006**	0.97	**0**.**011**	0.873
Coronary sinus Day 1	r		−0.066	**0**.**532**	0.061
	p		0.737	**0**.**001**	0.782
Femoral artery Day 1	r			0.112	0.179
	p			0.579	0.463
Femoral vein Day 1	r				0.233
	p				0.297

### Correlations between Galectin-3 levels and cardiac function

In the LVR group, we observed a reduction in the LVESV (32.66 ± 12.13 vs 44.69 ± 13.76, p = 0.02) and LVEDV (68.26 ± 20.50 vs 90.66 ± 25.3, p = 0.001) on day 1 compared to no remodelling group (Table 6). LVEF showed a decrease at day 180 (46.86 ± 5.80 vs 52.46 ± 7.34, p = 0.004; Table 6) in the LVR group compared to no remodelling group. One of the key parameters of LV diastolic function, E/E’, was significantly higher in the LVR group at six months (8.78 ± 2.97 vs 7.45 ± 1.71, p = 0.03; Table 6). We carried out comprehensive correlation analyses of all relevant echocardiographic parameters and Galectin-3 plasma levels, from different locations and time-points post-AMI. Galectin-3 in the median cubital vein on day 30 was correlated with a number of echocardiographic parameters of systolic and diastolic dysfunction at six months: positively with LVESV (r = 0.343, p = 0.03), diameter of LA (r = 0.446, p = 0.004), an increase in LVEDV (ΔLVEDV) (r = 0.571, p < 0.001), and an increase in LVESV (ΔLVESV) (0.579, p < 0.001), and negatively with LVEF (r = −0.509, p < 0.001) and a decrease in LVEF (ΔLVEF) (r = −0.599, p < 0.001; Table 7). A decrease in LVEF (ΔLVEF) was also negatively correlated with Galectin-3 plasma levels in the coronary sinus (r = −0.298, p = 0.02), femoral artery (r = −0.481, p = 0.001), femoral vein (r = −0.290, p = 0.03) on day 1, and LVEF at six months was negatively correlated with Galectin-3 plasma levels in the femoral artery on day 1 (r = −0.292, p = 0.049; Table 7). An increase in LVESV from day 1 to six months (ΔLVESV) was positively correlated with Galectin-3 levels in the femoral vein on day 1 (r = 0.29, p = 0.03; Table 7), and an increase in the LA diameter (ΔLA) between day 1 and six months, was also positively correlated with Galectin-3 levels in the femoral artery on day 1 (r = 0.331, p = 0.02; Table 7).

**Table 6:** Echocardiography parameters used to diagnose systolic and diastolic left ventricular dysfunction on day 1, 30 and 180, in patients with and without left ventricular remodelling, six months after acute myocardial infarction.

	No LVR (n = 35)	LVR (n = 22)	t* or Z**	p-value
Heart Rate (bpm)	75.09 ± 13.81	74.23 ± 15.45	0.218*	0.828
LVEDV 1 day	90.66 ± 25.3	68.26 ± 20.50	**3**.490*	**0**.**001**
LVEDV 30 day	90.45 ± 25.00	81.05 ± 33.63	1.154*	0.254
LVEDV 180 day	85.51 ± 27.50	89.55 ± 33.15	0.498*	0.620
LVESV1 day	44.69 ± 13.76	32.66 ± 12.12	**3**.333*	**0**.**002**
LVESV 30 day	39 (35.00–52.00)	36.50 (21.00–42.75)	1.554**	0.120
LVESV 180 day	42.00 ± 14.46	48.59 ± 18,07	1.519*	0.134
LVEF 1 day	51.49 ± 5.16	53.77 ± 6.35	1.489*	0.142
LVEF 30 day	52.41 ± 7.13	53.05 ± 7.07	0.318*	0.752
LVEF 180 day	52.46 ± 7.34	46.86 ± 5.80	**3**.027*	**0**.**004**
E/А 1 day	0.78 (0.64–1.13)	0.70 (0.56–0.74)	1.165**	0.244
E/А 30 day	0.87 (0.70–1.20)	0.78 (0.62–0.82)	0.028**	0.977
E/А 180 day	0.78 (0.70–1.20)	0.78 (0.62–0.87)	0.164**	0.869
E/Е′ 1 day	8.25 ± 2.97	8.13 ± 2.60	0.158*	0.875
E/Е′ 30 day	7.63 ± 1.78	8.78 ± 2.76	1.836*	0.072
E/Е′ 180 day	7.45 ± 1.71	8.78 ± 2.97	**2**.160*	**0**.**035**
LA 1 day	38.03 ± 5.56	37.91 ± 4.34	0.086*	0.932
LA 30 day	38.63 ± 4.95	37.85 ± 4.95	0.549*	0.585
LA 180 day	38.31 ± 4.89	40.00 ± 4.48	1.290*	0.203

All values are presented as mean ± SD or median with interquartile range (IQR).

Two-tailed unpaired t-test (normalized distribution; t(p)) or Man-Whitney (non-normalized distribution; Z (p)).

LVR – left ventricular remodeling.

**Table 7:** Correlation between plasma Galectin-3 levels and echocardiography parameters determined 6 months after acute myocardial infarction.

Variables		Aortic root Day 1	Coronary sinus Day 1	Femoral artery Day 1	Femoral vein Day 1	Median cubital vein Day 30
LVEDV 180 day	r	0.023	0.120	0.034	0.159	0.276
	p	0.860	0.383	0.823	0.232	0.090
LVESV 180 day	r	0.039	0.086	0.098	0.156	**0**.**343**
	p	0.769	0.522	0.516	0.241	**0**.**033**
LVEF 180 day	r	−0.164	−0.160	**−0**.**292**	−0.227	**−0**.**509**
	p	0.215	0.224	**0**.**049**	0.086	<**0**.**001**
LA 180 day	r	0.021	0.234	0.163	0.173	**0**.**446**
	p	0.876	0.094	0.280	0.194	**0**.**004**
ΔLVEDV	r	0.166	0.219	0.124	0.252	**0**.**571**
	p	0.209	0.102	0.412	0.057	<**0**.**001**
ΔLVESV	r	0.166	0.237	0.226	**0**.**290**	**0**.**579**
	p	0.210	0.076	0.131	**0**.**027**	<**0**.**001**
ΔLVEF	r	−0.149	**−0**.**298**	**−0**.**481**	**−0**.**290**	**−0**.**599**
	p	0.261	**0**.**024**	**0**.**001**	**0**.**027**	<**0**.**001**
ΔLA	r	0.244	0.068	**0**.**331**	0.108	0.274
	p	0.063	0.614	**0**.**025**	0.422	0.091

Δ – Change from Day 1 to day 180 or six months.

## Discussion

This is the first study which investigates the predictive biomarker value and dynamics of plasma Galectin-3 in the development of adverse LVR, six months post first AMI, using arterial and venous, central and peripheral blood collected on day 1 and 30 post-AMI. Here, we also provide extensive analyses of correlations between Galectin-3 levels at different time points and from different sampling locations after AMI, as well as parameters of cardiac function. Galectin-3 levels in the central and peripheral vein on day 1, and in the peripheral vein on day 30, following AMI, were positively associated with adverse LVR; whereas arterial Galectin-3 levels did not show any association with LVR. Positive correlation was also found between Galectin-3 levels in central and peripheral venous blood, as well as central arterial and central or peripheral venous blood, potentially suggesting myocardial synthesis of this marker. Galectin-3 plasma levels in the median cubital vein on day 30, demonstrated promising predictive value for the development of negative LVR, six months later, which was identified as an independent predictor of 1.55-fold increased risk of LVR when adjusted for age, diabetes and inflammatory markers.

LVR occurs in 10–35% of the patients after AMI^14^, often after an extensive STEMI infarction of the anterior wall, even after myocardial reperfusion is achieved by primary percutaneous coronary intervention (PCI)^15^. In our study LVR occurred in approximately 40% of the patients, which is higher than what is reported in other studies^16,17^ potentially due to higher percentage of patients with diabetes mellitus (35%). Other studies have investigated the role of Galectin-3 levels both in AMI and chronic heart failure, using experimental models of AMI and human studies^11,13,18,19^. We have also shown previously that Galectin-3 levels are elevated after NSTEMI AMI in patients with atrial fibrillation^20^, however, the predictive and mechanistic role of Galectin-3 in LVR post AMI is still in its infancy. In our study, patients who developed LVR, had higher levels of inflammatory markers (CRP and leukocytes count) at baseline, indicating the role of inflammation in LVR, likely followed by fibrosis; all of which are critical processes driving LVR^21,22^. Galectin-3′s role in inflammation and fibrosis has been well established^7,10^. Our results demonstrated general trend towards an increase in Galectin-3 secretion levels in the LVR group with the most predictive values being obtained at day 30 post AMI. Previous reports based on an animal MI model which reported that initial Galectin-3 secretion post-MI was mediated by interstitial cardiac macrophages^6^, prompted us to investigate Galectin-3 dynamics from different blood locations and in a timely manner. Published data from another animal model where Galectin-3 was knocked out demonstrated that post MI, low baseline levels of Galectin-3 were associated with bigger MI area, adverse remodelling and ventricular dysfunction, which are likely associated with reduced collagen deposition and macrophage infiltration^22^. This suggests that initial high levels of Galectin-3 post-AMI are protective, whereas prolongation of high levels of Galectin-3 are associated with cardiac fibrosis, which is associated with adverse LVR and clinical outcomes^12,23,24^. Therefore, measuring Galectin-3 levels in the AMI phase during early stages might not have a reliable prognostic value in terms of adverse LVR.

According to previously published studies, in patients with chronic heart failure, cardiac Galectin-3 expression is not differentially expressed compared to healthy controls, however it is increased in ischaemic myocardium^25^. This suggests that the role of Galectin-3 in LVR is more pronounced in previously healthy myocardium which becomes ischaemic following AMI. *Frunza and colleagues* demonstrated that Galectin-3 expression in healthy murine hearts is localized within macrophages and atrial cardiomyocytes; 7 days following exposure to increased intracranial pressure, Galectin-3 expression is noted in myofibroblasts also, and 28 days lates in ventricular cardiomyocytes^26^. In support of these findings, another study reported that the expression of Galectin-3 in cardiomyocytes was correlated with adverse LVR^27^.

In human studies investigating association between Galectin-3 levels and LVR post-AMI, conflicting results were reported. *Weir and colleagues* showed no clear association between Galectin-3 levels at baseline post-AMI and LVR, except in patients with preserved LVEF^12^ whereas *Di Tano and colleagues* reported adjusted odds ratio of 1.2 when Galectin-3 venous plasma levels were measured during the AMI phase, but with a lower predictive value when Galectin-3 was evaluated 30 days later^13^. In the latter study, diabetes was not identified as a risk factor in a univariate or multivariate analyses and the groups with and without LVR differed in terms of age and gender. In our study 59% of the patients in the LVR group had diabetes mellitus compared to 11.5% in Di Tano *et al*. study^13^. Also, our study included both NSTEM and STEMI patients whereas Di Tano *et al*. study included only STEMI patients^13^. Therefore, our cohort of patients was different in terms of the baseline risk factors. Nevertheless, our results are somewhat aligned showing, in a multivariate analysis, a 1.5-fold increase in the risk of LVR at six months based on Galectin-3 venous levels not during hospitalisation but 30 days after AMI, which is likely more reflective of the damaging effects of Galectin-3 rather than initial protective pro-inflammatory effects.

Furthermore, following out detailed analyses of correlations between Galectin-3 levels and cardiac function parameters over time, we demonstrated strong correlations between Galectin-3 plasma levels and several echocardiographic parameters which are used to assess LVR. No difference in average heart rate and frequency of arrhythmias between the two groups was observed during baseline echocardiography, indicating no influence of other factors on these parameters. Parameters of systolic dysfunction and adverse LVR at six months were associated with higher Galectin-3 concentration in the venous blood on day 1 whereas parameters of systolic and diastolic dysfunction were associated with higher Galectin-3 levels in the arterial blood on day 1. On day 30 higher Galectin-3 levels were associated with parameters of LVR, diastolic and systolic cardiac dysfunction at six months after AMI.

LVR is characterized by deterioration of LV function in systole, as well as in diastole. After AMI, a disorder of LV function in early diastole may develop in the form of impaired relaxation, and in the late diastole, filling pressure of the ventricle may be increased, due to the reduction in ventricular compliance. The reduced diastolic function represents one of the consequences of LVR syndrome after AMI^28^. According to our results, impaired LV function in diastole and increased filling pressure of LV in diastole is accompanied by increased Galectin-3 plasma concentration. Similarly, patients with LVR had lower LVESV and LVEDV on day 1. This group of patients, six months later had lower LVEF, possibly due to progression of the fibrosis and expansion of the infracted myocardial segment as reported before^29–31^. *Di Tano and colleagues* demonstrated similar results showing that LVEDV on admission was negatively associated with the risk of LVR 6 months post-AMI^13^.

### Limitations of the Study

Our study is unique as it measures Galectin-3 plasma levels in four different blood sampling locations including peripheral and central arterial or venous blood, and repeated sampling over time for venous peripheral blood. The main limitations of our study are the number of enrolled patients and limited 6-month follow-up period. While biomarkers of myocardial necrosis, troponin, and CKMB, were used to confirm the diagnosis of AMI, serial measurements of these biomarkers over time, to assess the extent of myocardial necrosis and correlation with Galectin-3 levels, were not performed. Also, as it was not ethically feasible to collect heart tissue samples in our study, we were unable to assess cardiac fibrosis or remodelling in these patients or to determine exactly the source of Galectin-3 secretion in relation to specific cardiac cell types.

In summary, we have demonstrated that Galectin-3 plasma levels in both central and peripheral venous blood on day 1 were increased in patients who developed LVR six months after AMI. No significant changes were observed in arterial blood. The most promising prognostic value was demonstrated with high levels of Galectin-3 in the cubital vein on day 30, which were independently associated with the 1.5-fold increased risk of LVR, six months after AMI. We have also demonstrated positive correlations between Galectin-3 concentration from different locations within arterial and venous blood and echocardiography parameters associated with diastolic and systolic dysfunction. Determining Galectin-3 plasma concentration at an appropriate time in patients post AMI, has both prognostic and therapeutic potential in identifying patients at risk of developing LVR.

## Methods

### Study participants

Fifty-seven patients experiencing a first AMI were enrolled in this study from December 2016 until November 2018. Non-invasive and invasive diagnostic procedures, pharmacotherapy, as well as myocardial revascularization by PCI, were performed in accordance with the institutional guidelines and the International Cardiology Associations recommendations. The patients who initially had severely impaired renal function (GFR < 30 ml/min), systemic inflammatory diseases, cancer, or other valid reasons preventing their participation in this study, were excluded. The written informed consent was obtained from all participants prior to their inclusion in the study. The research was performed in accordance with the Helsinki Declaration and approved by the Ethics Committees of the Faculty of Medicine in Nis and Clinical Centre “Kragujevac”.

### Variables of interest

In all patients with AMI, blood sampling was carried out within the first 24 h, during PCI. Galectin-3 concentration was measured on day one in central and peripheral arterial blood i.e. in the aortic root and the femoral artery, and in the central and peripheral venous blood i.e. in the right atrium near the coronary sinus and the femoral vein, and on day 30, in the cubital vein, following AMI (Supplementary Fig. 2). Plasma was separated from the whole blood by centrifugation at 3,000 g for 10 min at 25 °C, aliquoted and frozen at −80 °C. Commercially available ELISA kit (BGM, Inc., Waltham, MA, USA) was used to determine Galectin-3 plasma levels according to the manufacturer’s instruction.

Echocardiography was performed on day 1, 30 and 180 or six months following AMI and the following parameters were evaluated: LVEDV, LVESV, LVEF (biplane area-length echocardiography method using Simpson’s formula^26^), ratio of mitral flow velocity in early and late diastole (E/A), the ratio of mitral flow velocity and mitral annulus velocity in early diastole (E/E’), and left atrium (LA) diameter. There are no clear recommendations for the diagnosis of LVR, previously reported studies had a variety of definitions, and in our study LVR was defined as an increase in LVESV ≥ 20% six months following AMI which separated patients with definitive diagnosis of LVR. Physicians who performed echocardiographic assessments were blinded to the biomarker results.

Routine blood tests were performed within the first 24 h of AMI. In addition to routine clinical parameters, the levels of hsCRP and proBNP were also measured. Biomarkers of myocardial necrosis, troponin and CKMB, were quantified to confirm the diagnosis of AMI. Serial testing of these biomarkers over time, to assess the extent of myocardial necrosis, was not performed.

### Statistical analysis

The obtained data were processed using the Statistical Package for Social Sciences (SPSS, v. 21.0; Chicago, IL, USA). Continuous variables were presented as the mean value with standard deviation or median with interquartile range, and categorical variables as absolute numbers of cases with the percentage. The differences between two groups were tested using Student’s t-test or Mann-Whitney U-test, depending on the normality of the continuous data distribution, or using the χ2-independence test with Yates’s correction for continuity of categorical variables. The correlation between two continuous variables was assessed based on the Pearson’s correlation coefficient. The influence of putative risk factors on dichotomous outcome was examined by stepwise multivariate logistic regression analysis with backward elimination of each insignificant variable (p ≥ 0.1), and the results were expressed as adjusted odds ratios with respective 95% confidence intervals. The diagnostic value of Galectin-3 concentrations in different blood sampling locations at different time points were investigated by constructions of the ROC curves. The statistical significance was determined as p < 0.05.

## Supplementary information

Supplementary Figure 1

Schematic of the study events.
