# Supplementary material for: Association between Galectin-3 levels within central and peripheral venous blood, and adverse left ventricular remodelling after first acute myocardial infarction
Source: Sci Rep. 2019 Sep 11;9:13145. doi: 10.1038/s41598-019-49511-4 (PMC6739356; doi:10.1038/s41598-019-49511-4)

Association between Galectin-3 levels within central and peripheral venous blood, and adverse left ventricular remodelling after first acute myocardial infarction

Olivera M. Andrejić[a], Rada M. Vučić[b,c], Milan Pavlović[d,i], Lana McClements[f], Dragana Stokanović[g], Tatjana Jevtović –Stoimenov[h], Valentina N. Nikolić[g]

[a]Clinic for Pulmonary Diseases, Clinical Centre Kragujevac, Zmaj Jovina Street 30, 34000 Kragujevac, Serbia

[b]University of Kragujevac, Serbia, Faculty of Medical Sciences, Department of Internal medicine, Svetozara Markovica Street 69, 34000 Kragujevac, Serbia

[c]Clinic for Cardiovascular Diseases, Clinical Centre Kragujevac, Zmaj Jovina Street 30, 34000 Kragujevac, Serbia

[d]Department of Internal Medicine - Cardiology, Medical Faculty, University of Nis, Bulevar dr Zorana Djindjica 81, Nis, Serbia

[i]Clinic for Cardiovascular Diseases, Clinical Centre Nis, Bulevar dr Zorana Djindjica 48, Nis, Serbia

[f]School of Life Sciences, Faculty of Science, University of Technology Sydney, Sydney, PO Box 123 Broadway, NSW 2007, Australia

[g]Department of Pharmacology and Toxicology, Medical Faculty, University of Nis, Bulevar dr Zorana Djindjica 81, Nis, Serbia

[h]Institute of Biochemistry, Medical Faculty, University of Nis, Bulevar dr Zorana Djindjica 81, Nis, Serbia

Declarations of interest: None.

Financial support: This study funded by Faculty of Medicine, University of Nis, (Grant No INT-MF19), as well as Serbian Ministry of Education, Science and Techno-logical Development (Grant No III41018 and 44044).

***Reprints and correspondence**: Dr Rada M. Vučić; 00381631123610, email: rada.vucic@gmail.com; Milovana Gusica 8/15, 34000 Kragujevac, Serbia

Supplementary Figure 1: ROC curves for predicting the risk of developing adverse left ventricular remodelling based on Galectin-3 concentrations in the aortic root (AUC 0.607, p=0.180) (1A), femoral artery (AUC 0.618, p=0.197) (1B), right atrium near coronary sinus (AUC 0.691, p=0.02) (1C), and femoral vein (AUC 0.661, p=0.044) (1D).

1A. Aortic root

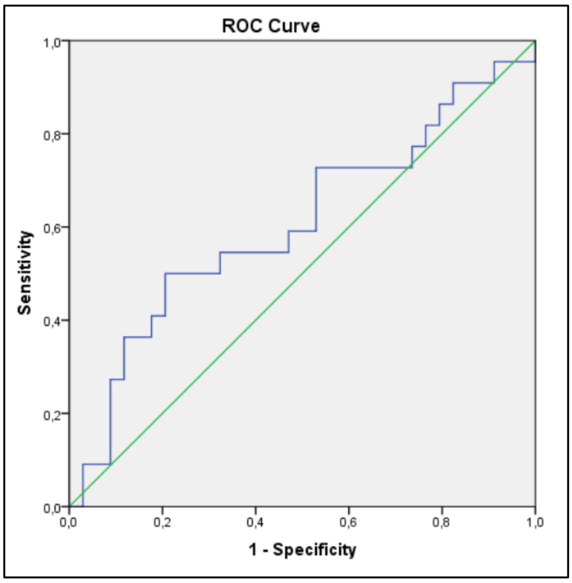

1B. Femoral artery

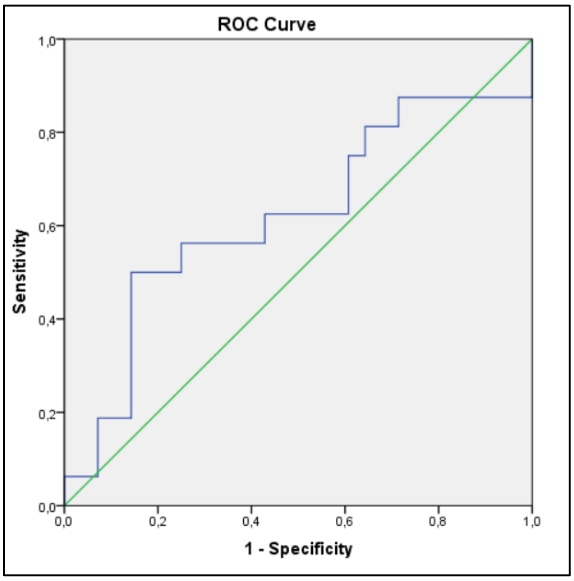

1C. Right atrium near Coronary sinus

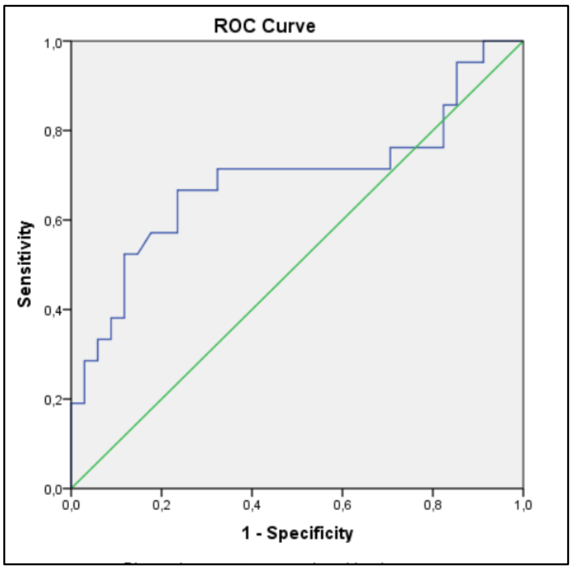

1D. Femoral vein

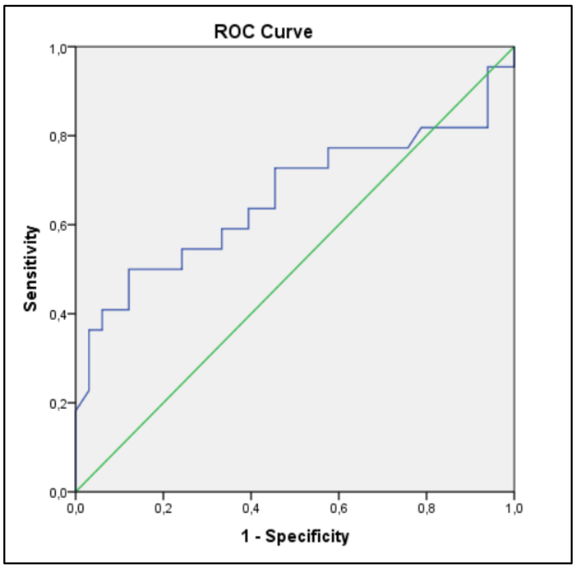

Supplement: Supplementary file 1 — Supplementary Figure 1 [file 41598_2019_49511_MOESM1_ESM.pdf]
