# Supplementary material for: Association between Galectin-3 levels within central and peripheral venous blood, and adverse left ventricular remodelling after first acute myocardial infarction
Source: Sci Rep. 2019 Sep 11;9:13145. doi: 10.1038/s41598-019-49511-4 (PMC6739356; doi:10.1038/s41598-019-49511-4)

**Association between Galectin-3 levels within central and peripheral venous blood, and adverse left ventricular remodelling after first acute myocardial infarction**

Olivera M. Andrejić[a], Rada M. Vučić[b,c], Milan Pavlović[d,i], Lana McClements[f], Dragana Stokanović[g], Tatjana Jevtović –Stoimenov[h], Valentina N. Nikolić[g]

[a]Clinic for Pulmonary Diseases, Clinical Centre Kragujevac, Zmaj Jovina Street 30, 34000 Kragujevac, Serbia

[b]University of Kragujevac, Serbia, Faculty of Medical Sciences, Department of Internal medicine, Svetozara Markovica Street 69, 34000 Kragujevac, Serbia

[c]Clinic for Cardiovascular Diseases, Clinical Centre Kragujevac, Zmaj Jovina Street 30, 34000 Kragujevac, Serbia

[d]Department of Internal Medicine - Cardiology, Medical Faculty, University of Nis, Bulevar dr Zorana Djindjica 81, Nis, Serbia

[i]Clinic for Cardiovascular Diseases, Clinical Centre Nis, Bulevar dr Zorana Djindjica 48, Nis, Serbia

[f]School of Life Sciences, Faculty of Science, University of Technology Sydney, Sydney, PO Box 123 Broadway, NSW 2007, Australia

[g]Department of Pharmacology and Toxicology, Medical Faculty, University of Nis, Bulevar dr Zorana Djindjica 81, Nis, Serbia

[h]Institute of Biochemistry, Medical Faculty, University of Nis, Bulevar dr Zorana Djindjica 81, Nis, Serbia

**Declarations of interest:** None.

**Financial support:** This study funded by Faculty of Medicine, University of Nis, (Grant No INT-MF19), as well as Serbian Ministry of Education, Science and Techno-logical Development (Grant No III41018 and 44044).

***Reprints and correspondence**: Dr Rada M. Vučić; 00381631123610, email: rada.vucic@gmail.com; Milovana Gusica 8/15, 34000 Kragujevac, Serbia

Supplementary Figure 2. Schematic of the study events.

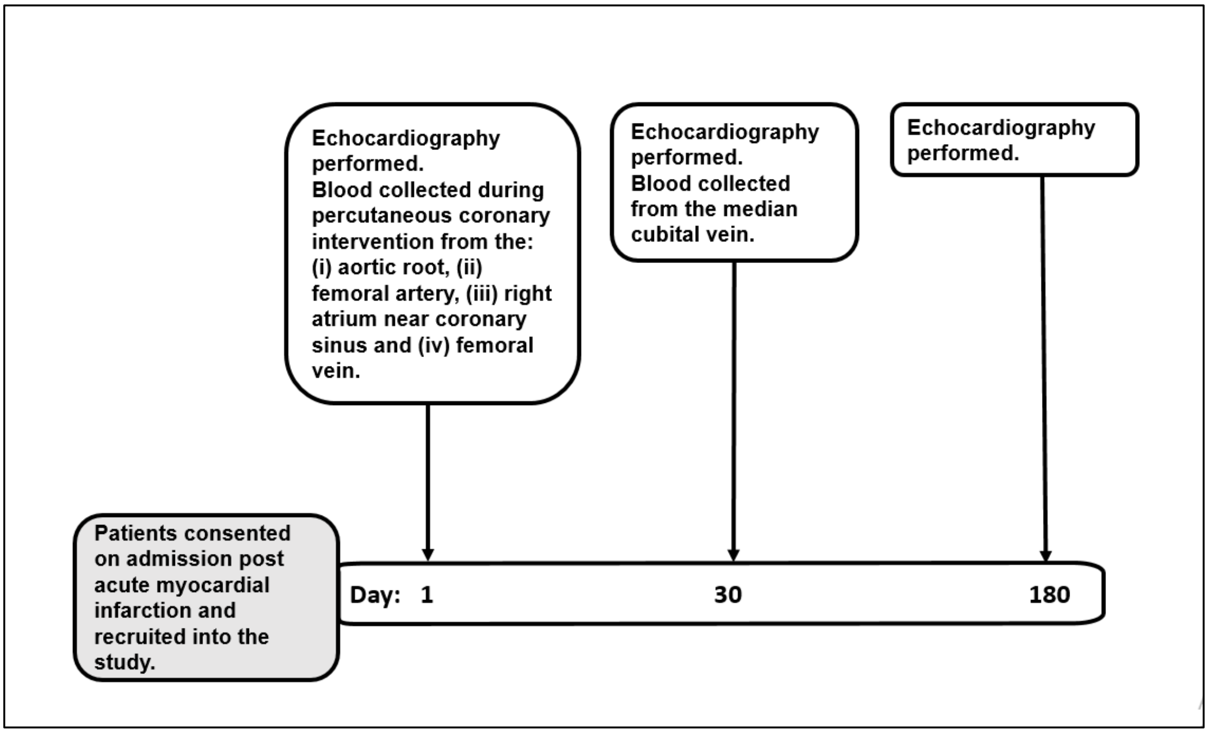

Supplement: Supplementary file 2 — Schematic of the study events. [file 41598_2019_49511_MOESM2_ESM.pdf]
